# Detection of a Recurrent TMEM38B Gene Deletion Associated with Recessive Osteogenesis Imperfecta

**DOI:** 10.15190/d.2021.3

**Published:** 2021-03-31

**Authors:** Khushnooda Ramzan, Maha Alotaibi, Rozeena Huma, Sibtain Afzal

**Affiliations:** ^1^Department of Genetics, King Faisal Specialist Hospital and Research Centre, Riyadh, Saudi Arabia; ^2^Department of Genetics, Children's Hospital, King Saud Medical City, Riyadh, Saudi Arabia; ^3^King Faisal Specialist Hospital and Research Centre, Riyadh, Saudi Arabia; ^4^College of Medicine, Alfaisal University, Riyadh, Saudi Arabia

**Keywords:** Osteogenesis imperfecta, mutations, recessive.

## Abstract

Osteogenesis imperfecta is a clinically and genetically group of heterogeneous disorders associated with decreased bone density, brittle bones, bone deformity, recurrent fractures, and growth retardation. Osteogenesis imperfecta is commonly associated with mutations of the genes encoding for type I collagen (COL1A1/COL1A2). Mutations in other genes, some associated with type I collagen post-translational processing, have also been identified as the cause of osteogenesis imperfecta. Mutations in the transmembrane protein 38B (TMEM38B) gene have been reported in a rare autosomal recessive form of osteogenesis imperfecta.  TMEM38B encodes TRIC-B - a trimeric intracellular cation channel type B which is essential to modulate intracellular calcium signaling. In this study, we are reporting a case of osteogenesis imperfecta type XIV from a Saudi consanguineous family. Our patient was an eight-month-old child with short limbs, club feet, and lower limb deformities with developmental delay. Radiological findings were consistent with the evidence of osteogenesis imperfecta. There was no evidence of impaired hearing or blue sclera and based on the clinical assessment, we classified our patient as a non-syndromic osteogenesis imperfecta. A pathogenic deletion in the chromosome 9q31.2 region, partially encompassing the TMEM38B gene, was detected using chromosomal microarray analysis. This study expands our knowledge about the rare type of osteogenesis imperfecta in our consanguineous population. Besides, it emphasizes the use of genomic medicine in clinical practices to formulate early interventions to clinically improve the patient’s condition.

## INTRODUCTION

Osteogenesis imperfecta, also known as “brittle bone disease”, is a hereditable connective tissue disease. It is a rare disorder with a prevalence of 4-21 per 100,000 births (Martin and Shapiro, 2007). Osteogenesis imperfecta is characterized by decreased bone mass, increased bone fragility, multiple fractures, short stature, and other complications^1-3^. Extra-skeletal manifestations of osteogenesis imperfecta include impaired hearing, blue sclera, dentinogenesis imperfect, macrocephaly, joint hyperlaxity, neurological complications, and cardiopulmonary defects^4^. The phenotypic spectrum of osteogenesis imperfecta cases ranges from mild to lethal, where typically, the *de novo* osteogenesis imperfecta cases are severe and lethal, and familial cases are less severe. Osteogenesis imperfecta is genetically heterogeneous, and many genetic causes have been identified underlying the phenotype. The classical Sillence types of osteogenesis imperfecta (types I-IV), which comprise about 80-85% of cases, are inherited in an autosomal dominant manner caused by defects in the genes that encode type I collagen, *COL1A1,* and *COL1A2*^3^. The rare forms of osteogenesis imperfecta (types V-XVIII), mostly with an autosomal recessive mode of inheritance, are caused by mutations in genes encoding proteins involved in post-translational modification or folding of type I collagen. The genes described in association with autosomal recessive osteogenesis imperfecta include *CRTAP*, *P3H1*, *PPIB*, *SERPINH1*, *FKBP65*, *SERPINF1*, *BMP1*, *WNT1*, and *FKBP10*^5,6^.

A study of multiplex consanguineous families and simplex cases identified a homozygous deletion of *TMEM38B *exon 4 in three Saudi families with a moderately severe bone phenotype characterized as osteogenesis imperfecta type XIV (MIM# 615066). The identified deletion resulted in a premature stop codon and a truncated protein (c.455_542del; p.Gly152Alafs*5)^7^. This loss of function allele of *TMEM38B* was also identified in three Bedouin Israeli consanguineous families with autosomal recessive osteogenesis imperfecta^8^.

**Figure 1:**
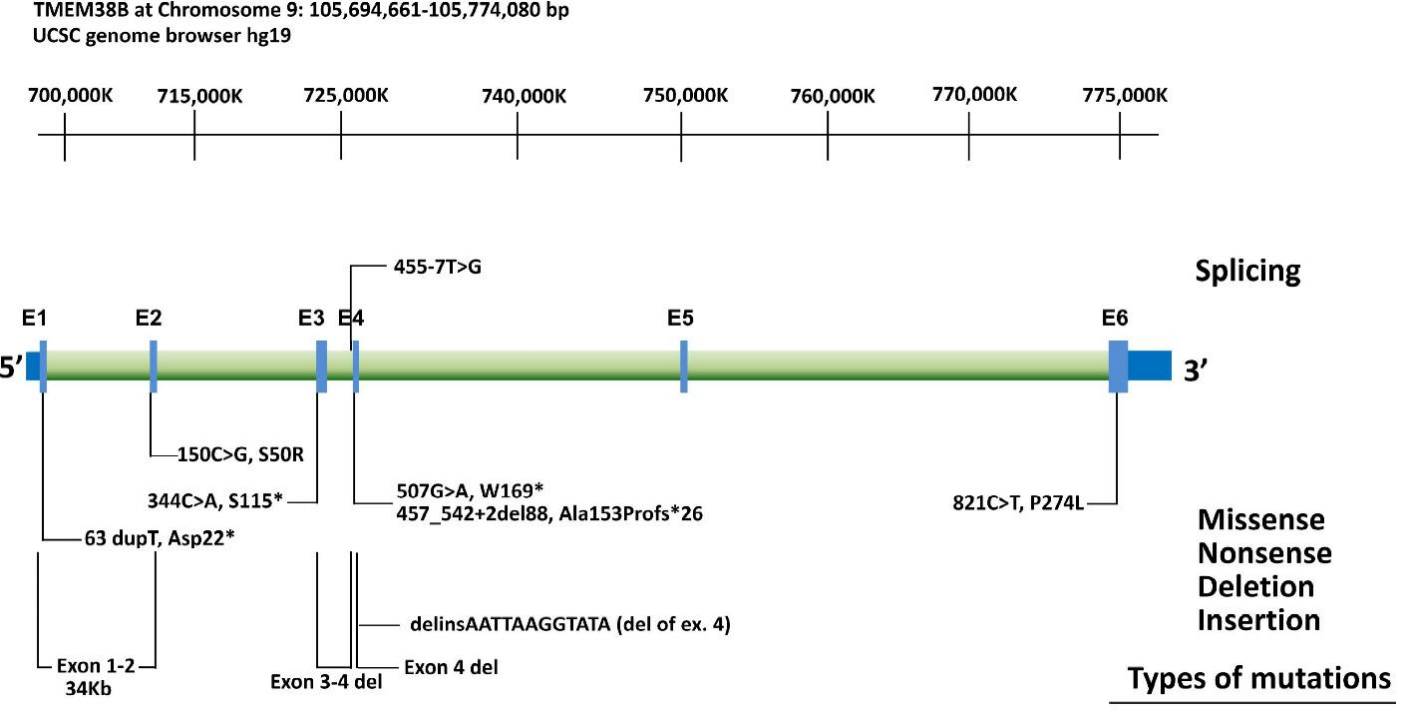
TMEM38B mutations associated with osteogenesis imperfecta type XIV

*TMEM38B* encodes endoplasmic reticulum membrane monovalent cation-specific channel which is involved in the release of calcium (Ca^2+^) from intracellular stores. TMEM38B, also known as TRIC-B, is one of the two trimeric intracellular cation (TRIC) channels. TRIC-B deficiency causes bone disease due to defective Ca^2+ ^release and signaling in the bone cells^9^. The role of encoded TRIC-B in intracellular Ca^2+^ homeostasis and Ca^2+^ flux kinetics in the endoplasmic reticulum supports that the absence of TMEM38B is associated with osteogenesis imperfecta due to dysregulation of collagen biosynthesis^10^. Tric-A encoded by Tmem38a, is dispensable for the normal development and fertility in mouse, whereas Tmem38b knockout mouse died shortly after birth due to birth asphyxia^11,12^. Regardless of the ubiquitous pattern of expression for *TMEM38B*, the reported phenotype for the mouse knockout was limited to the alveolar pathology and abnormal Ca^2+^ signaling in the cardiomyocytes, thereby the abnormal development of bone as a result of Tmem38b deficiency in mouse remains inexplicable^11,12^. *TMEM38B* mutations associated with osteogenesis imperfecta type XIV are shown in Figure 1.

## CASE REPORT

**Figure 2:**
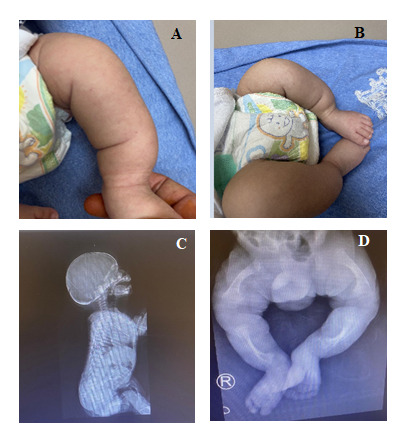
Clinical features of the patient with TMEM38B related phenotype **A. **and **B. **Images of the lower extremities showing mild shortening and bowing. Bilateral talipes equinovarus deformity was observed;** C. **and** D. **Representative radiograms of a patient with *TMEM38B *at 8 months of age. Skull radiographs showing generalized osteopenia, thin cortex of calvarium, and thickening of the occipital part of the skull. Patent sutures with no evidence of Wormian bone. Lateral spine view of the whole body showing generalized osteopenia and normal shape and height of vertebrae with maintained disc space. Chest showing osteopenia, thin ribs, mild widening of the costochondral junctions of the dorsal ribs and decreased aeration of both lungs. Right upper limb radiograph showing opteopenia. Anteroposterior view of lower limbs showing significant bilateral bowing and femoral deformity. The bowing was more prominent in the lower extremities in a background of metaphyseal irregularity. Diffuse osteopenia and healed fracture of the distal left femoral metaphysis were noted.

An eight-month-old male child was referred to the genetic clinic with a history of having short limbs, club feet, and bilateral leg bowing (lower limb deformities) with developmental delay (Figure 2).

Detailed clinical data, which included growth, development, and bone fracture history were investigated. The patient is a product of full-term normal vaginal delivery with no antenatal, post-natal, or neonatal complications with a birth weight of 2.6 kg. His APGAR score was normal. Parents are a consanguineous couple, his mother had no history of abortion, or stillbirth and his father had Down syndrome. The family history was non-contributory as there was no history of bone fractures in other family members. At presentation, he had an abnormal facial shape, with no other dysmorphic facial features. He had a height of 64 cm (-2 SD), and a weight of 7.8 kg (50° percentile). His head circumference was 44 cm (50° percentile). He was not able to sit, stand, or crawl. He had no signs of abnormal hearing, sclera, teeth, or skin. He had Talipes equinovarus deformity bilaterally (Figure 2B). Other systemic examinations and investigations were unremarkable and within normal ranges. Abdominal and brain ultrasound were also unremarkable. Cardiac echocardiogram was normal. He suffered from a femoral fracture at the age of 7 months. Through whole-body radiological assessment was performed, and the findings were consistent with the evidence of osteogenesis imperfecta. Representative radiographic images of the affected patient are shown in Figure 2C, Figure 2D.

## GENETIC STUDIES

Peripheral blood samples (5 ml) were collected in EDTA tubes from the index case and parents. Written informed consent was obtained before the samples’ collection. Chromosomal microarray analysis (CMA) was performed by Centogene using CentoArrayCyto™ - HD incl. SNP test. CMA method enables the detection of copy number variations (CNVs) associated with chromosomal imbalances in the nuclear genome and for the detection of absence or loss of heterozygosity (AOH/LOH) and regions of homozygosity (ROH). CMA technology is limited only to detect large genomic copy number imbalances and AOH in the nuclear genome. Balanced chromosomal rearrangements such as balanced inversions, reciprocal translocations, and inversions are not detectable. CMA cannot detect imbalances in the mitochondrial genome, repeat sequences, segmental duplications, complete uniparental heterodisomy for the entire chromosome, point mutations and indels, low levels of mosaicism, or CNVs in the genomic regions that are not represented on the microarray.

Genomic DNA was extracted using the DNA Extraction Kit (QIAamp DNA; Qiagen, Frankfurt, Germany), following the manufacturer’s instructions. Briefly, 250 ng of genomic DNA was fragmented, amplified, and then hybridized to the array. The Cytoscan HD array contains 2.7 million markers, including 750,000 single nucleotide polymorphism (SNP) markers, across the whole genome covering 96% of the genes, thereby enabling the detection of CNVs/large deletions/duplications. The obtained results were analyzed using the Chromosome Analysis Suite (ChAS, Affymetrix) and interpreted with the Database of Genomic Variants (http://dgv.tcag.ca/) and Decipher database.

Cytoscan HD array detected a homozygous 22-kb deletion on long arm of chromosome 9 (9q31.2) with base-pair coordinates 108,484,180_108,506,650 [GRCh37]. This identified deletion resulted in partial loss of both copies of exon 4 of the *TMEM38B* gene and has been classified as pathogenic according to the recommendations of the Centogene and American College of Medical Genetics and Genomics (ACMG) guidelines (Table 1). The father of our patient had Down syndrome, while the genetic diagnosis of Down syndrome was not established for this patient as no clinically relevant CNV was found in chromosome 21. Interestingly, only a few confirmed parenting cases by fathers with Down syndrome have been described in the literature. Down syndrome's theoretical risk in the offspring is 50% until more pregnancies fathered by Down syndrome males are reported^13,14^.

**Table 1:** Identified TMEM38B gene deletion CNV – copy number variation; *According to ISCN 2016; **according to ChAS Affymetrix, ***according to ACMG 2011, modified.

CNV DESCRIPTION*	SIZE (kb)	RefSeq GENES**	INTERPRETATION***	PATIENT RELEVANT PHENOTYPE
arr [GRCh37] 9q31.2(108484180_108506650)x0	22	TMEM38B	Pathogenic	Osteogenesis imperfecta, type XIV (AR)

## DISCUSSION

Osteogenesis imperfecta is a connective tissue disorder and is characterized by low bone mass and bone fragility. Classical classification of osteogenesis imperfecta subtypes had been suggested based on clinical features and considerable phenotypic variability. The majority of osteogenesis imperfecta cases are autosomal dominant with underlying mutations in one of two genes encoding for type I collagen alpha chains, COL1A1 and COL1A2^2^. Most of the affected individuals with osteogenesis imperfecta have significant physical disabilities and require multidisciplinary clinical management and genetic analysis.

Shaheen et al. reported twelve individuals from three families with autosomal recessive osteogenesis imperfecta type XIV, characterized by variable degrees and severity of multiple fractures and osteopenia. None of the reported patients had teeth abnormalities, hearing loss, or blue sclera. In all of the affected patients, fractures first occurred either prenatally or by six years of age^7^. Within a critical interval on chromosome 9q31.1-31.3, a biallelic homozygous deletion of exon 4 of the *TMEM38B* gene was found in all the affected members^7^. Volodarsky et al. identified the same deletion in the exon 4 and low *TMEM38B* mRNA levels in three southern Israeli Bedouin consanguineous families^8^. A biallelic 35-kb deletion encompassing exon 1 and 2 of *TMEM38B­*, was identified in an 11-year-old girl with osteogenesis imperfecta^15^. Later three children affected with osteogenesis imperfecta from Chinese Hans families were reported with *TMEM38B­ *pathogenic mutations (c.455-7T>G in intron 3 and c.507G>A; p.(Trp169Term) in exon 4). The efficacy of bisphosphonates and zoledronic acid on the bones of those patients was also prospectively observed.

In this study, we are reporting another case of autosomal recessive osteogenesis imperfecta, type XIV, from our region. The genetic diagnosis of the autosomal recessive osteogenesis imperfecta type XIV is confirmed by detecting a 22-kb homozygous loss on 9q31.2 encompassing the *TMEM38B* gene. The phenotype of our patient is not different from the reported range of phenotypes ascribed to this clinically heterogeneous disorder in the previous reports. Our clinical assessment based on the absence of blue sclera and hearing impairment suggests that our index case represents the non-syndromic form of osteogenesis imperfecta. The fracture frequency in patients reported by Shaheen et al. varied and improved with age after puberty. Similarly, in our patient being 10 months old, we anticipate the improvement with the age. The genotype-phenotype comparison of patients from Shaheen et al. 2012^7^ with our patient is summarized in Table 2.

**Table 2:** Phenotype-Genotype comparison of 3 patients from Shaheen et al. 20127 with our patient

Family ID	#3	#11	#13	Our case
Age (years old)	2.8-16	2.8-16	2.8-16	10 months
Sex				Male
Consanguineous	+	+	+	+
Family History	+	+	+	-
No. of affected in the family	4	4	2 (identical twins)	1
Onset of presentation	6 years	2.8-16 years	Prenatal	8
Dysmorphic features				Abnormal facial features
Teeth (abnormal)	-	-	-	-
Blue sclera,	-	-	-	-
Progressive hearing loss	-	-	-	-
Other organ involvement	-	-	-	-
Multiple fractures	+ (Variable degree of severity)	+ (Less frequent)	+ (Variable degrees of severity)	-
Osteopenia	+	+	+	+
TMEM38B mutation/ chromosomal microarray analysis	TMEM38B (NM_018112.1) Complete exon 4 deletion c.455_542del; p.(Gly152Alafs*5) homozygous	TMEM38B (NM_018112.1) Complete exon 4 deletion c.455_542del; p.(Gly152Alafs*5) homozygous	TMEM38B (NM_018112.1) Complete exon 4 deletion c.455_542del; p.(Gly152Alafs*5) homozygous	arr[GRCh37] 9q31.2 (108484180 108506650)x0

This study strengthens the fact that abnormal bone development is associated with *TMEM38B* deficiency leading to autosomal recessive osteogenesis imperfecta in humans and further helps us to elucidate the pathogenesis of *TMEM38B* pathogenic variants. Osteogenesis imperfecta cases in our region have significant genetic heterogeneity and this additional case highlights the contribution of* TMEM38B* Bedouins’ founder mutation to the genetic landscape of autosomal recessive osteogenesis imperfecta in our population. In addition, it would help in genetic counseling of the family as well as for future family planning.
